# Prosthetic Valve Infective Endocarditis Secondary to Neisseria elongata

**DOI:** 10.7759/cureus.35609

**Published:** 2023-02-28

**Authors:** Mena Henien, Farrah Malik, Sharjeel Ahmad

**Affiliations:** 1 Department of Internal Medicine, University of Illinois Chicago College of Medicine, Peoria, USA

**Keywords:** septic emboli, dental infection, next generation sequencing (ngs), prosthetic valve infective endocarditis, neisseria elongata

## Abstract

Neisseria is a large genus of bacteria consisting of organisms colonizing mucosal tracts of many animals. *Neisseria elongata* is a unique member, given that it is a Gram-negative rod, unlike others which are diplococci. Unlike most Neisseria species, *N. elongata *is catalase-negative and Superoxol-negative. These unique characteristics of *N. elongata *can make it more difficult to identify. Although considered to be a commensal of the nasopharyngeal tract, this organism has increasingly been identified as a cause of significant disease in humans including endocarditis. We present a case report and literature review of *N. elongata *causing prosthetic valve endocarditis.

## Introduction

Neisseria is a genus consisting of organisms colonizing mucosal tracts of many animals. *Neisseria elongata* is a unique member, given that it is a Gram-negative rod, unlike others which are diplococci [1]. Although considered to be a commensal of the nasopharyngeal tract, this organism has increasingly been identified as a cause of significant disease in humans [2].

## Case presentation

A male patient in his early 60s with a past medical history of hypertension, chronic kidney disease stage 2, and mitral valve prolapse requiring mitral valve annuloplasty three years earlier presented with a two-week history of headache and dizziness. His symptoms progressed to severe chills, and night sweats, then one week prior to admission, he developed more severe bi-temporal headaches and “brain fog.” His wife noticed he was intermittently confused and slow to respond. He denied focal weakness, numbness, blurry vision, fevers, or gait changes. He reported a broken tooth while eating a few weeks prior, but denied dental pain or infection. He received prophylactic antibiotics prior to all dental procedures with the last procedure being two years ago.

He lived on a farm and was a retired carpenter who enjoyed hunting. He handled a deer carcass in the field one day prior to symptom onset. He was transferred from an outlying hospital due to concerns for stroke. On admission, his temperature was 100.3°F, blood pressure was 104/73 mmHg, heart rate was 86 beats/minute, respiratory rate 23 breaths/minute, and oxygen saturation was 96%. Examination revealed poor dentition but no signs of active periodontal disease, with no heart murmur, signs of cardiac failure, or peripheral stigmata of endocarditis. Complete blood count was unremarkable. The following labs were abnormally elevated: c-reactive protein 18.5 mg/dL, aspartate aminotransferase 144 U/L, alanine aminotransferase 187 U/L, alkaline phosphatase 151 U/L, and creatinine 1.4 mg/dL.

**Figure 1:**
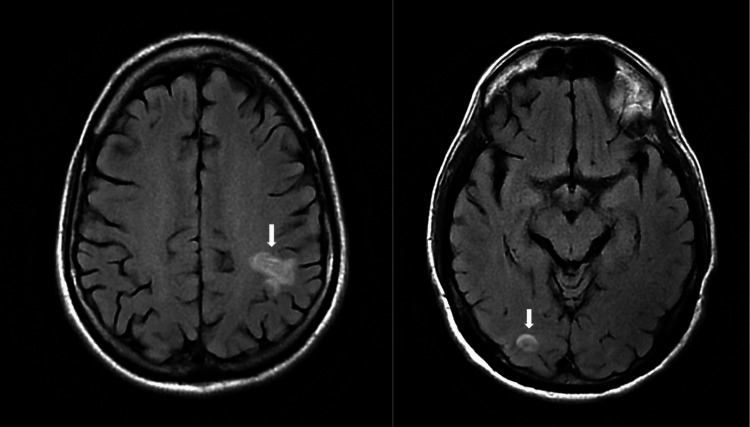
MRI brain axial FLAIR demonstrating septic emboli (arrows). MRI: magnetic resonance imaging; FLAIR: fluid-attenuated inversion recovery

**Figure 2:**
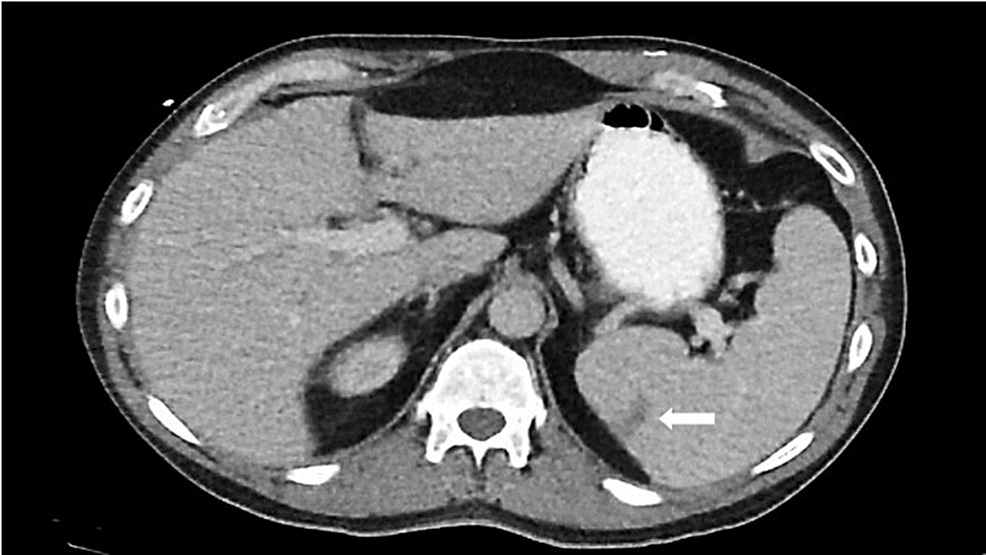
CT abdomen with contrast showing splenic infarct (arrow).

**Figure 3:**
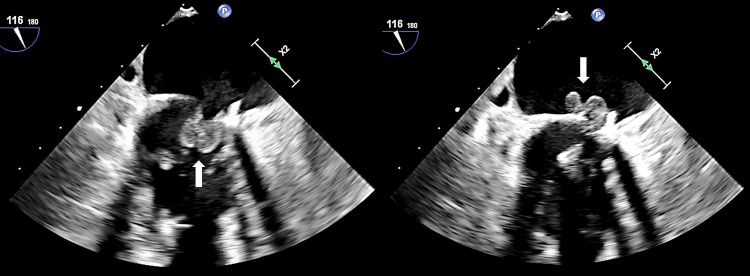
TEE showing 1.6x0.8 cm multi-lobulated vegetation during systole and diastole (arrows). TEE: transesophageal echocardiogram

Magnetic resonance imaging (MRI) brain showed multifocal regions of acute ischemic infarcts in a pattern consistent with showering septic emboli (Figure 1). Computerized tomography (CT) of the abdomen showed a wedge-shaped splenic lesion compatible with a possible small infarct (Figure 2). Transthoracic echocardiography (TTE) showed unchanged mitral valve thickening. Transesophageal echocardiography (TEE) was positive for large 1.6 cm, multi-lobulated, freely mobile echo-density involving the mitral annuloplasty ring, with moderate mitral regurgitation in a pattern suggestive of valve leaflet perforation (Figure 3).

**Figure 4:**
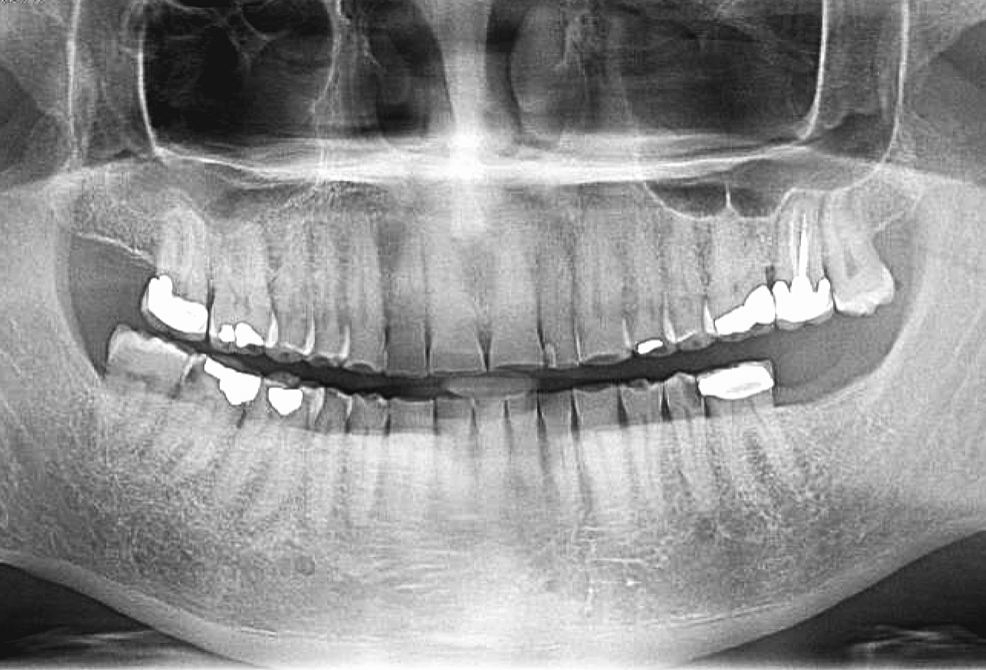
Panoramic x-ray showing no evidence of dental infection.

Blood cultures acquired on admission showed presence of Gram-negative bacilli 20 h after collection. These were initially identified as *Moraxella catarrhalis* (Gram-negative diplococci) after four days. This diagnosis was questioned due to the discrepancy between the Gram stain and the organism identified. Given the severity of the disease in this patient with infectious endocarditis and embolic phenomenon, further investigations were needed for accurate diagnosis. Blood next-generation sequencing of microbial cell-free DNA (NGS cfDNA) (“Karius test,” Karius, Redwood City, CA) was ordered and showed the presence of *N. elongata *at 6192 DNA molecules/μL [3]. Blood cultures were then sent from the outlying hospital to a reference lab (Quest Diagnostics, Secaucus, NJ) for verification which confirmed *N. elongata*. Repeat cultures after initiation of antimicrobial therapy were negative. Since *N. elongata *is part of oropharyngeal flora, panoramic x-rays were obtained which showed no evidence of dental infections (Figure 4).

The patient was empirically started on piperacillin-tazobactam 4.5 g every 8 h and switched later to ceftriaxone 2 g every 24 h IV. He was scheduled to complete six weeks of therapy. Cardiothoracic surgery service was consulted which recommended nonsurgical management.

He developed neutropenia, dizziness, and a burning sensation in his mouth, nose, and throat, necessitating cessation of ceftriaxone on day 40 instead of day 42 (six weeks). He was seen in the cardiothoracic surgery clinic four weeks after the completion of treatment and was found to be asymptomatic.

## Discussion

*N. elongata *is a Gram-negative rod, known to be part of oropharyngeal flora, with a biochemical profile similar to Kingella and Moraxella species making initial identification difficult. It is different from the other members of the genus Neisseria as it is rod-shaped organism. Subspecies of *N. elongata *include elongata, nitroreducens, and glycolytica. Endocarditis caused by *N. elongata *has been reported to cause acute febrile illness, large destructive lesions, and systemic complications as seen in our patient. There are several cases reported in the literature of *N. elongata *infective endocarditis, most of which are involving native cardiac valves. To the best of our knowledge, only six previous cases with prosthetic valve endocarditis have been reported, as noted in Table 1. Five of these cases involved the aortic valve and one case of bi-valvular (aortic and mitral) prosthetic valves [4].

**Table 1:** Cases of prosthetic valve infective endocarditis secondary to Neisseria elongata.

Reference	Age/gender	Valve	Valve risk factors	Likely source	Antibiotic regimen	Outcome
Sawas et al. in 2015 [4]	55/M	Aortic and mitral	Prosthetic	Unclear	Ceftriaxone (6 weeks) after surgical intervention	Full recovery
Dominguez and Smith in 1998 [5]	50/M	Aortic	Prosthetic	Unclear	Ampicillin (4 weeks), after surgical intervention	Unclear
Brandao et al. in 2021 [6]	65/M	Aortic	Prosthetic valve/Bentall procedure	Unclear	Antibiotic tailored to culture sensitivities (specific antibiotics not mentioned) (8 weeks)	Full recovery/no recurrence at 1-year follow-up
Evans et al. in 2007 [7]	70/M	Aortic	Prosthetic valve	Unclear	Ceftazidime 1 g bid/gentamicin (5 days), amoxicillin/gentamicin (3 weeks), ceftriaxone (3 additional weeks)	Full recovery
Picu et al. in 2003 [8]	79/M	Aortic	Prosthetic valve	Dental abscess	Ceftriaxone/gentamicin (2 weeks), ceftriaxone (4 additional weeks)	Full recovery
Meuleman et al. in 1996 [9,10]	74/F	Aortic	Prosthetic	Dental abscess	Gentamicin (14 days), ampicillin (6 weeks)	Full recovery

Dental infections or recent dental instrumentation are thought to be the most common risk factor. Per our literature review, only two of the six cases had clear dental infections (abscesses) at the time of diagnosis. While some of the cases showed poor oral hygiene, they did not have an obvious dental infection and no other clear source was identified.

Infectious endocarditis caused by *N. elongata*, although rare, is usually aggressive, causing severe valvular damage, and likely necessitating surgical intervention for both native and prosthetic valves. Two (33.3%) of the prosthetic valve infections mentioned in the table (Table 1) required replacement of the prosthetic valves [4,5].

In addition to our case, two of the six previously reported cases (33.3%) were found to have septic embolic phenomenon including brain and splenic infarcts [4,6]. *N. elongata *is typically sensitive to cephalosporins and aminoglycosides and is variably susceptible to penicillin. Most of these cases were treated with ampicillin or a third-generation cephalosporin such as ceftriaxone. Gentamicin was used in the initial phase of treatment in some cases [7-10]. Antibiotic therapy duration ranged from six to eight weeks, with the exception of one case who received four weeks of therapy after surgical valve replacement [5].

Although rare, *N. elongata *should be on the differential list of organisms when suspecting infective endocarditis. Given the unique Gram stain characteristics of this organism compared to the rest of the Neisseria genus members, it can be misidentified. Further testing, such as blood next-generation sequencing of microbial cell-free DNA (NGS cfDNA), or other methods could be helpful for further identification. During the hospitalization of our patient, the outside hospital microbiology lab took a long time to identify the organism. The infectious diseases consultant recommended blood NGS cfDNA testing to expedite the identification of the organism. Oropharyngeal and ear infections were considered as possible sources; however, no identifiable source of the infection was found. The discrepancy between the identification of organisms by the outlying hospital lab and NGS cfDNA testing led to the sample being sent to a reference lab which confirmed the results of NGS cfDNA testing. In addition, the overall clinical picture was more consistent with *N. elongata *rather than *Moraxella catarrhalis* infection.

Early diagnosis is essential provided how aggressive the infection is and the systemic complications associated with it. When infection by *N. elongata *is suspected, further dental history should be explored since this is recognized as the most common source, and dental imaging should be considered. Additionally, patients with prosthetic cardiac valves need to be counseled on the importance of maintaining good oral hygiene and discussing antibiotic prophylaxis prior to dental procedures with their primary care providers.

## Conclusions

*N. elongata *is a member of the Neisseria genus with unique characteristics distinguishing it from the rest of this genus. It is a Gram-negative rod, that is catalase-negative and superoxol-negative. These features often cause misidentification of the organism during the routine workup and blood cultures. *N. elongata* is a common colonizing organism of the nasopharyngeal tract yet has been identified to cause significant disease in humans including destructive endocarditis lesions. These lesions have been associated with systemic complications, such as septic embolic phenomenon and septic shock. Given how aggressive this infection is, early diagnosis is the key to appropriate management. Further dental history is recommended since this appears to be the most common source of infection. Besides routine blood cultures, additional workup such as next-generation sequencing methods is often required to correctly identify this organism.
